# MEG Assessment of Expressive Language in Children Evaluated for Epilepsy Surgery

**DOI:** 10.1007/s10548-019-00703-1

**Published:** 2019-03-20

**Authors:** Elaine Foley, J. Helen Cross, Ngoc J. Thai, A. Richard Walsh, Peter Bill, Paul Furlong, Amanda G. Wood, Antonella Cerquiglini, Stefano Seri

**Affiliations:** 1 Aston Brain Centre, School of Life and Health Sciences, Aston University, Birmingham, UK; 2 Children’s Epilepsy Surgery Service, Birmingham Children’s and Women’s Hospital NHS Foundation Trust, Birmingham, UK; 3 Developmental Neurosciences Programme, Institute of Child Health, University College London, London, UK; 4 Department of Paediatric Neurology, Great Ormond Street Hospital NHS Foundation Trust, London, UK; 5 CRICBristol, Bristol’s Clinical Research and Imaging Centre, Bristol, UK; 6 Developmental Neuropsychiatry Section, Dipartimento di Scienze e BiotecnologieMedico-Chirurgiche, “Sapienza” Universita’ di Roma Polo Pontino, Latina, Italy; 7 Brain and Mind, Clinical Sciences, Murdoch Children’s Research Institute, Melbourne, Australia

**Keywords:** Children, Magnetoencephalography, Language lateralization, Hemispheric dominance, Beamformers, Functional mapping, Epilepsy surgery

## Abstract

Establishing language dominance is an important step in the presurgical evaluation of patients with refractory epilepsy. In the absence of a universally accepted gold-standard non-invasive method to determine language dominance in the preoperative assessment, a range of tools and methodologies have recently received attention. When applied to pediatric age, many of the proposed methods, such as functional magnetic resonance imaging (fMRI), may present some challenges due to the time-varying effects of epileptogenic lesions and of on-going seizures on maturational phenomena. Magnetoencephalography (MEG) has the advantage of being insensitive to the distortive effects of anatomical lesions on brain microvasculature and to differences in the metabolism or vascularization of the developing brain and also provides a less intimidating recording environment for younger children. In this study we investigated the reliability of lateralized synchronous cortical activation during a verb generation task in a group of 28 children (10 males and 18 females, mean age 12 years) with refractory epilepsy who were evaluated for epilepsy surgery. The verb generation task was associated with significant decreases in beta oscillatory power (13–30 Hz) in frontal and temporal lobes. The MEG data were compared with other available presurgical non-invasive data including cortical stimulation, neuropsychological and fMRI data on language lateralization where available. We found that the lateralization of MEG beta power reduction was concordant with language dominance determined by one or more different assessment methods (i.e. cortical stimulation mapping, neuropsychological, fMRI or post-operative data) in 89% of patients. Our data suggest that qualitative hemispheric differences in task-related changes of spectral power could offer a promising insight into the contribution of dominant and non-dominant hemispheres in language processing and may help to characterize the specialization and lateralization of language processes in children.

## Introduction

Identification of language-relevant cortex in pediatric age is one of the most challenging aspects of the pre-surgical evaluation process for frontal and temporal lobe epilepsies, further complicated by the high incidence of atypical language organization in patients with a history of drug-resistant epilepsy (Duchowny et al. 1996; Hamberger and Cole 2011). A range of non-invasive tools and methodologies to assess language dominance have been developed with the ultimate aim of replacing the Intracarotid Amobarbital Procedure (IAP), a procedure accepted for decades as the gold-standard diagnostic test in adults (Abou-Khalil 2007). When applied to pediatric age, the reliability of the proposed methods is influenced among others by the time-varying effects of the epileptogenic lesion and of the on-going seizures on maturational phenomena (Holmes 2016). Furthermore, the tolerability of currently available diagnostic procedures can make the assessment of hemispheric dominance for language and interpretation in children particularly challenging (de Ribaupierre et al. 2012).

Functional MRI is the most widely used of the minimally/non-invasive techniques for presurgical language mapping in children (Rodin et al. 2013), but MEG is gaining increasing acceptance (Pang et al. 2011). Compared to MRI, methods based on electromagnetic measures of brain activity have the advantage of being insensitive to the distortive effects of anatomical lesions on brain microvasculature or metabolism on the developing brain (Demonet et al. 2005) and also provide a less intimidating recording environment for younger children (Pang et al. 2011). Initial reports on MEG language localization have shown encouraging results with a wide variety of cognitive paradigms (Pirmoradi et al. 2010) and analysis strategies (Edwards et al. 2010; Hirata et al. 2010; McDonald et al. 2010; Papanicolaou et al. 2004). The suitability of MEG language mapping protocols in adults as an alternative to the Wada procedure have been addressed with validation studies conducted with various approaches and methodologies and overall high concordance rates (Bowyer et al. 2005; Findlay et al. 2012; Hirata et al. 2004; Maestu et al. 2002; Merrifield et al. 2007; Papanicolaou et al. 2004; Tanaka et al. 2013).

Adult MEG studies using equivalent current dipole models or distributed source models to localize evoked activity have reported good concordance (75–90%) with findings from the IAP (Breier et al. 2001; Doss et al. 2009; McDonald et al. 2010; Papanicolaou et al. 2004). More recently, task-related time-varying changes in the electromagnetic power spectrum have been used to detect hemispheric dominance during language tasks and have also demonstrated high concordance with IAP ranging from 85 to 95% (Findlay et al. 2012; Hirata et al. 2004; Kim and Chung 2008). A study of the relationship between MEG localization of language-sensitive areas and electrical stimulation mapping in two adults (Simos et al. 1999a) added convergent evidence that MEG is a useful, non-invasive method for assessing hemispheric language dominance in adults with refractory seizures who are surgical candidates. Yet despite the overall positive contribution of MEG in the assessment of language localization in typically developing individuals, data on its clinical use in clinical pediatric populations is still relatively modest. Recent studies have reported the feasibility of passive paradigms in children and adolescents under sedation (Rezaie et al. 2014) and during sleep (Van Poppel et al. 2012), but MEG studies using more traditional expressive language tasks in pre-surgical assessment of children are still limited.

The aim of this study was to provide a contribution to addressing this gap by measuring hemispheric lateralization of MEG oscillatory activity during awake expressive language processing in pediatric age in a naturalistic pre-surgical setting. We investigated feasibility and reliability of pre-operative MEG assessment of language lateralization in 28 pediatric patients using a verb generation task.

## Methods

### Participants

Twenty-eight pediatric patients with drug-resistant epilepsy under evaluation for resective surgery participated in the study. Ten male and eighteen females (mean age 12 years; age range 7–18 years) were included in the study (see Table 1 for a summary of patient demographics). All patients were referred to the Wellcome Laboratory for MEG studies at the Aston Brain Centre in Birmingham from the two largest centers participating in the Children’s Epilepsy Surgery Service (CESS) program of NHS England between 2013 and 2016 for localization of the irritative zone and eloquent cortex mapping. Twenty patients were referred from Birmingham Women’s and Children’s Hospital NHS Foundation Trust and eight from Great Ormond Street Hospital for Children NHS Foundation Trust. Inclusion criteria included being native English speakers and the ability to perform the task during a practice session prior to the MEG recording. Informed consent was obtained from all patients prior to MEG recording and it was established from seizure diary sheets that all patients were seizure-free for at least 24 h prior to recording. The study was conducted according to the ethical principles of the declaration of Helsinki and approved by the UK NHS research ethics committee (Ref. 17/WM/0174).

**Table 1:** Summary of patient characteristics and results

Pt.	Age (years), Gender	Age at Onset (y)	Handedness	Etiology	Seizure lateralization	Surgical procedure	Language lateralization: MEG	Language lateralization: neuropsychology and fMRI	Language lateralization: DCS
1	7,M	2	R	TSC	L	L ATL	LI = 29.35, L	L	L
2	8,M	5	R	FCD	L	L ATL+	LI = 19.82, L	L	L
3	9,F	1	R	MTS	L	L ATL+	LI = 21.39, L	L	L
4	7,F	5	R	FCD	L	L ATL	LI = 60.35, L	L	L
5	8,F	3	R	MTS	L	L ATL+	LI = 26.03, L	L	L
6	8,M	1	R	FCD	L	L ATL+	LI = 33.02, L	L	L
7	11,F	1	R	MTS	L	L ATL+	LI = 58.62, L	L	L
8	10,F	1	R	MTS	L	L ATL+	LI = 28.09, L	L	L
9	14,M	2	R	Ganglioma	R	R Temporal Lesionectomy	LI = 76.31, L	L	\
10	16,F	2	R	FCD	L	L ATL+	LI = 52.43, L	L	\
11	17,F	1	L	Stroke	L	L Hemispherotomy	LI = − 47.91, R	R	\
12	17,F	10	R	FCD	R	R ATL	LI = 38.66, L	L	\
13	16,F	10	R	FCD	L	L ATL	LI = 64.95, L	L	L
14	16,F	13	L	FCD	L	L ATL+	LI = − 80.62, R	L	\
15	15,F	2	L	FCD	L	No Surgery	LI = 4.77, Bilat	Bilat	\
16	8,M	6	L	FCD	L	L Hemispherotomy	LI = − 51.26, R	R	\
17	15,F	3	R	TSC	L	R Frontal Lesionectomy	LI = − 34.93, R	L	\
18	12,M	1	L/Ambi	MTS	L	L ATL	LI = − 13.46, Bilat	Bilat	\
19	18,M	4	R	MRI Neg	R	No Surgery	LI = 36.51, L	L	\
20	11,M	8	L	FCD II	L	L ATL	LI = 98.02, L	L	\
21	14,F	1	R/Ambi	FCD	L	No Surgery	LI = 61.77, L	L	\
22	16,M	10	R	FCD Ib	R	R Frontal Lesionectomy	LI = 63.68, L	L	L
23	17,F	7	R	MRI Neg	L	No Surgery	LI = 33.81, L	L	\
24	11,M	2	R	FCD	R	No Surgery	LI = 86.17, L	L	\
25	10,F	5	R	MRI Neg	L	No Surgery	LI = 86.83, L	Bilat	\
26	12,F	2	R	MRI Neg	R	R Temporal Resection	LI = 42.78, L	L	\
27	16,F	11	R	MRI Neg	L	No Surgery	LI = 81.76, L	L	\
28	15,F	5	R	FCD Ia	R	R Frontal & Parietal Resection	LI = 30.26, L	L	\

*L* left, *R* right, *Ambi* ambidextrous, *Bilat* bilateral, *ATL* anterior temporal lobectomy, *ATL* + anterior temporal lobectomy + hippocampoamygdalectomy, *TSC* Tuberous Sclerosis Complex, *FCD* focal cortical dysplasia, *MTS* mesial temporal sclerosis, *MRI Neg* MRI negative, *LI* lateralization index

### Experimental Paradigm

We used a ‘child-friendly’ verb generation task described in a previous study by our group (Fisher et al. 2008). In this task participants were visually presented with a series of single nouns and asked to generate an associated verb for each (e.g. ‘BALL’—‘throw’/’catch’). To separate out component processes involved in articulation and associated muscle artefacts, participants were instructed to initially generate responses covertly and then to vocalize their response on presentation of a visual cue. The overt component of the task was included to determine that participants were performing the task correctly. The task commenced with a three-second ‘passive’ phase where participants were asked to focus on a fixation cross. Then a noun was visually presented and participants were instructed to silently generate their response (three second ‘active’ phase). This was followed by an image of Mr. Chatterbox (Copyright © 2018 THOIP - a Sanrio company) cueing participants to verbalize their response. The Mr. Chatterbox© image remained on the screen for 3 s followed by an inter-stimulus interval of 500 ms before the onset of the next trial. Sixty data trials were collected for this task. Visual stimuli were presented using Presentation software (Neurobehavioral Systems, Inc.). Fixation was monitored with a camera system positioned in front of the patient with on-line tracking of eye movement. Epochs with changes in fixation point greater than 1 cm were discarded from the analysis. The stimuli were projected using a projector located outside of the shielded room and projected on to a translucent screen, which was positioned 85 cm from the participant subtending a 3-degree visual angle. High-resolution anatomical MRI scans (3D inversion recovery whole-head volume sequences were acquired with 1mm^3^ isotropic resolution) previously acquired within a 3-month interval at the two referring centers as part of the presurgical evaluation, were used for co-registration with the MEG data.

### MEG Data Acquisition

MEG data were recorded in a magnetically shielded room using an Elekta-Neuromag TRIUX whole-head system (Helsinki, Finland) with 204 planar dc-SQUID gradiometers and 102 magnetometers. Participants were seated in an upright position in the MEG scanner. Data were acquired with 2 KHz sampling rate, and low-pass filter of 660 Hz. One bipolar EEG channel was dedicated to recording ECG. Five coils were placed on the patient’s head, three on the front and one on each mastoid for continuous monitoring of head position. To allow the translation between the MEG coordinate system and the patient’s structural MRI, three head position fiducial points at the nasion, left and right pre-auricular points were digitized with a Polhemus Fastrack device, which was also used to measure the surface shape of each participant’s head and to localize the electromagnetic head coils with respect to that surface. Each participant’s head shape file was then extracted and co-registered with the high-resolution anatomical MRI sequence. Co-registration was performed using in-house software based on an algorithm designed to minimize the squared Euclidean distance between the Polhemus and the MRI surfaces. The accuracy of this procedure has been shown to be within 5 mm (Adjamian et al. 2004).

### MEG Data Analysis

Artefacts were removed from the raw data with MaxFilter software (Elekta Neuromag Oy, version 2.2.10) that uses the temporal extension of signal space separation (tSSS) (Taulu and Hari 2009). Max-filtered data were visually inspected to identify “bad channels” that were removed if present. The data were then analyzed in the Matlab R2012a environment (The MathWorks Inc., Natick, MA.) using the FieldTrip toolbox (Oostenveld et al. 2011). Responses to the overt segment of each trial were initially inspected for accuracy and for missed responses. Data analysis was then performed on the correct covert trials only; 5 s epochs were created based on 2.5 s pre- and 2.5 s post-stimulus. Data epochs of interest were then visually inspected for any additional artefacts caused by muscle activity or SQUID jumps and any contaminated trials were discarded. Time windows with relatively long duration were chosen to capitalize on the sustained nature of task-induced spectral changes and differentiate these effects from transient responses related to stimulus delivery.

### Whole Brain Analysis

Individual head shapes were created for each patient based on their own structural MRI and were co-registered to the MEG coordinate system. Realistic, single-shell brain models were also constructed for each participant based on their structural MRIs (Nolte 2003). An MEG source-space voxel size of 0.5 cm was used to compute the lead field matrix. An adaptive spatial filtering beamforming technique in the frequency-domain, known as dynamic imaging of coherent sources (DICS), was used to determine sources of neuronal activity associated with the verb generation task (Gross et al. 2001). Spectral perturbation data in the source space were produced for the beta frequency range (13–30 Hz). The choice of frequency-band was selected based on previous evidence demonstrating spectral power changes within this range during language processing (Findlay et al. 2012; Fisher et al. 2008; Hirata et al. 2010; Pang et al. 2011). At the individual level, statistical differences between the active and baseline conditions were calculated using a paired-sample t test. A nonparametric randomization test was then used to correct for multiple comparisons (Maris and Oostenveld 2007). Source estimates were interpolated onto the individual anatomical images and results are displayed on individual patient’s T1 MRIs. Source estimates were subsequently normalized to a standard MNI template brain for illustrative purposes only.

Lateralization index (LI) was computed based on t-values of the event related power decrease in Brodmann areas (BA) 6, 44, 45 and 22 in the left (t_L_) and right hemisphere (*t*_R_). These regions of interest were selected based on the volumes of interest used in previous clinical MEG studies of language (Findlay et al. 2012; Hirata et al. 2004) and they represent areas that are commonly explored with direct cortical stimulation, including Broca (BA44/45) and Wernicke’s (BA22) areas (Ojemann et al. 2003). The t-values of the most prominent decrease in oscillatory power within the 13–30 Hz frequency range within the related region and its contralateral homologous region were selected.

The index used was:

$$LI=100*\frac{{\left( {{t_L} - {t_R}} \right)}}{{\left( {|{t_{L~}}|+|{t_R}|} \right)}}$$

A positive value indicated greater power decrease in left hemisphere and a negative value in the right. The use of lateralization indices in neuroimaging studies has received some criticism as they are sensitive to user defined thresholds (Chlebus et al. 2007; Jansen et al. 2006; Rutten et al. 2002) and rely upon decisions about the choice of total or peak strength within regions of interest (Fisher et al. 2008). However, the lateralization index can be a useful measure to address the problem of excessive false positives in language studies where bilateral activation is common (Findlay et al. 2012; Papanicolaou et al. 2004). Here it was used as an additional quantitative measure to complement the qualitative assessment of spectral power analysis.

### Regions of Interest Analysis

The output of the beamformer has been referred to as ‘virtual electrode’ or ‘virtual sensor’ which can be visualized as time–frequency representations of activity arising from specific voxels where spectral power changes are identified (Singh et al. 2003) and used to characterize in greater detail frequency-specific spectral power changes associated with the task (Hillebrand et al. 2005). Virtual electrodes were constructed based on the data covariance matrix using a 5 s window from 2.5 s pre-stimulus to 2.5 s post stimulus onset, with a bandwidth of 1–30 Hz. The difference in source estimates between pre-stimulus baseline and task-related activity was evaluated, in specific regions of interest corresponding to Brodmann areas 6, 44, 45 and 22.

Time–frequency analysis on the virtual sensor signal was performed using EEGLab software version 11.0.0.0b (Delorme and Makeig 2004) running in Matlab R2012a. Single-trial epochs were analyzed using a moving window short-time Fourier transform with 200 overlapping time windows per trial. The average log-power in the baseline period was subtracted from the log-power at each time–frequency point in the active period, yielding the measure conventionally known as “event-related spectral perturbation,” or ERSP (Makeig 1993). The color at each image pixel in the spectrogram then indicates power (in dB) at a given frequency and latency relative to the stimulus. Significance of deviations from baseline power was assessed using a bootstrap method, where a surrogate data distribution was constructed by selecting spectral estimates for each trial from randomly selected latency windows in the baseline period and then averaging them. This process was applied 200 times to produce a surrogate ‘baseline’ amplitude distribution whose specified percentiles were then used as significance thresholds, with p < .05 after correcting for multiple comparisons using the FDR method.

### Comparison Modalities

#### Neuropsychological Testing and fMRI

Full neuropsychological testing was performed on all patients as part of their routine surgical workup and included measures of handedness, language competence and IQ. These were assessed according to standardized administration of age-appropriate measures. Intellectual ability was assessed with the Wechsler Abbreviated Scale of Intelligence (WASI, Elliott 1990; Wechsler 1999) which includes measures of verbal and non-verbal abilities and gives rise to a measure of full scale IQ (FSIQ). Verbal fluency was assessed using the Delis-Kaplan Executive Function System (D-KEFS; Delis et al. 2001) and vocabulary was assessed using the British Picture Vocabulary Scale (BPVS3; Dunn et al. 2009).

Functional MRI was performed to assess language function in 15 of the patients as part of their presurgical workup. Functional MRI studies were performed on a 3 T Siemens TIM Trio with a gradient echo imaging sequence. A whole-head echo-planar acquisition protocol was used to measure blood oxygen level-dependent (BOLD) changes during the verb generation task. The BOLD sequence was acquired with the following parameters: TR 3000 ms; TE 30 ms; voxels 3 × 3 × 3 mm; field of view 192 mm; 44 contiguous 3 mm thick axial slices. A full volume 1 mm^3^ high resolution anatomical MRI was acquired using an inversion recovery sequence in the sagittal plane to co-register with the patient’s fMRI data.

Data were pre-processed and analysed using Statistical Parametric Mapping software (SPM12; http://www.fil.ion.ucl.ac.uk/spm/). Data for each participant were pre-processed using slice time correction, followed by realignment where the mean image was used as reference. Data were normalised to the standard SPM12 MNI EPI template and a 6 mm^3^ full width by half maximum (FWHM) isotropic gaussian kernel was used to spatially smooth the normalised images. Statistical analysis at the individual level was estimated using the general linear model and the LI Toolbox (Wilke and Schmithorst 2006) was used to compute language lateralisation indices in the inferior frontal gyrus (Broca’s area) and superior temporal gyrus (Wernicke’s area). In line with previous studies, patients with indices > 0.2 were considered left-dominant and patients with indices < 0.2 were considered to show atypical (right or bilateral) language dominance (Berl et al. 2014).

### Direct Cortical Stimulation

Direct Cortical Stimulation was performed in 10 out of 28 patients according to an established method (Jayakar et al. 1992). Contiguous electrodes pairs were stimulated using 5 s duration trains of square-wave current pulses of alternating polarity, 0.3 ms duration, with a frequency of 50 Hz. Stimulus intensity was increased by 1 mA steps until either one of the following was observed: after-discharge on the intracranial EEG; a clinical response with speech arrest, or an electrographically-documented seizure.

## Results

**Fig. 1:**
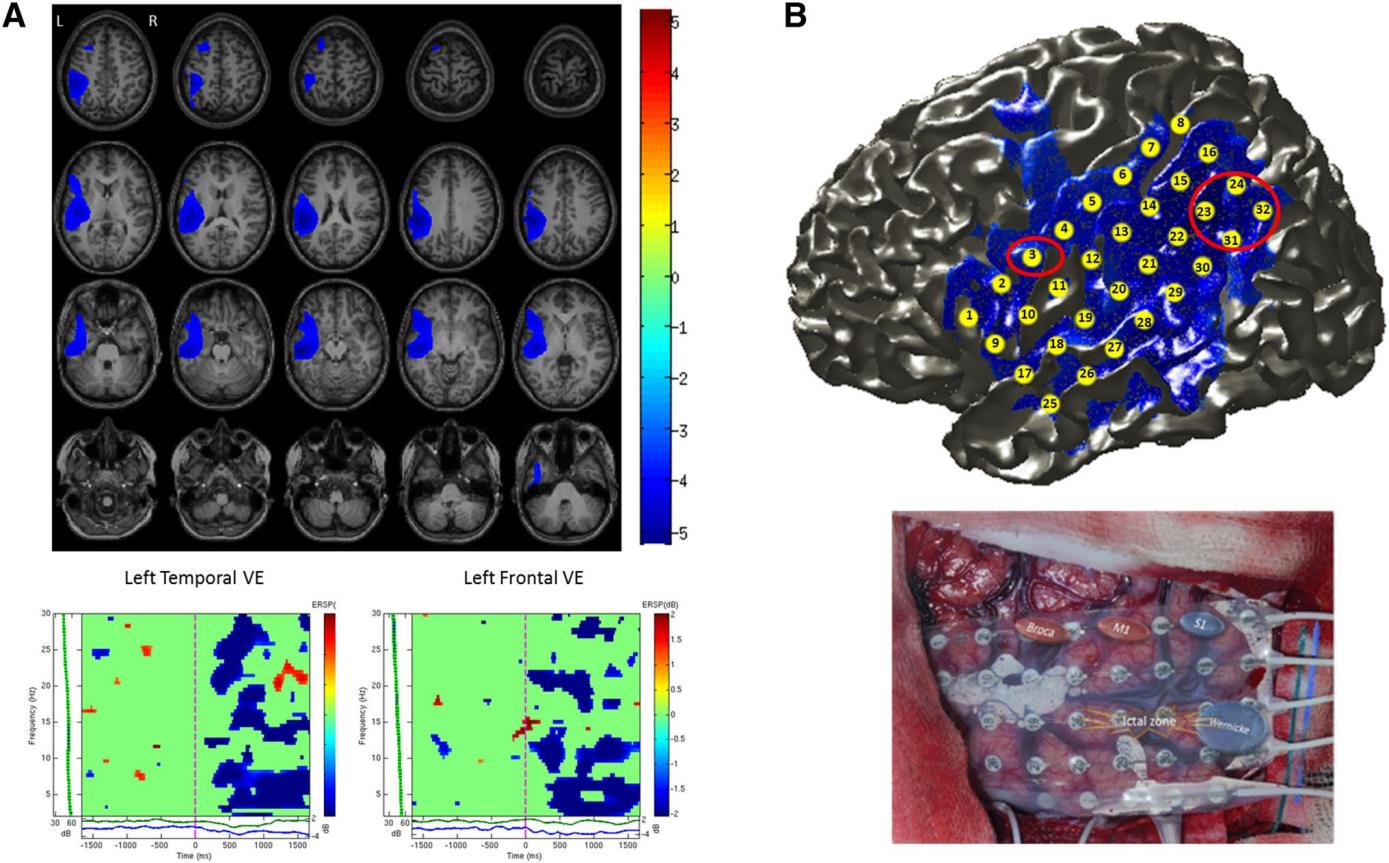
**a** Beamformer source localization results of verb generation task for an individual patient (Pt. 13) are shown overlaid on axial slices of the patient’s T1 MRI. Sources showing significant decreases in beta oscillatory power (13–30 Hz) from 2.5 s pre- to 2.5 s post-stimulus onset were identified in left temporal and left frontal regions (LI = 64.95). Time–frequency analysis of a virtual electrode (VE) constructed in the left temporal lobe shows an early sustained decrease in beta power during covert verb generation. Similarly, time–frequency analysis of a virtual electrode constructed in the left frontal lobe shows decreases in beta power from around 200 ms during covert verb generation. **b** Top: Electrode grid co-registered and overlaid on cortical surface of the patients MRI along with MEG beamformer source localization results. Red circles indicate location of Wernicke’s and Broca’s areas as identified during direct cortical stimulation. The peak source of decreased beta power (13–30 Hz) localized by MEG corresponds to Wernicke’s area in this patient. Bottom: Photograph of 32-channel grid shows results from cortical stimulation, where Broca’s area was mapped to contact 3 and Wernicke’s area to contacts 23–24 and 31–32. When these contacts were stimulated the patient had difficulty producing verbs, was confused and stuttered

All of the patients performed the task successfully, with at least 95% of the trials correctly completed. This was assessed on the basis of their overt responses to the stimuli. The verb generation task was associated with significant decreases in beta oscillatory power (13–30 Hz) in frontal and temporal lobes. While the topography of maximum spectral power decrease varied at the voxel level across patients, its location was in the superior temporal gyrus (STG) and inferior frontal gyrus (IFG), typically extending to include areas of the precentral gyrus and premotor cortex. The power decrease generally commenced 250–500 ms after noun presentation and was sustained for approximately 1000 ms. In most patients, the response was sustained throughout the whole active phase of the task (e.g. see Fig. 1).

The lateralization of power reduction in the beta frequency band was concordant with language dominance determined by one or more assessment methods (i.e. cortical stimulation mapping, neuropsychological, fMRI or post-operative data) in 89% of patients (see Table 1). None of the patients who proceeded to surgery displayed any language deficits compared to their pre-surgical status at one-year follow up. In all twenty-eight cases the MEG data were compared with other available presurgical non-invasive data including neuropsychological and fMRI data on language lateralization where available. Ten right-handed patients had direct cortical stimulation mapping and the results of the MEG analysis was concordant with both the non-invasive and invasive data, confirming left hemisphere lateralization in all ten patients (see Table 1). Figure 1 provides an illustrative example of an individual patient (Pt. 13) showing good concordance between the non-invasive MEG language lateralization data and invasive cortical stimulation mapping. MEG sources of decreased beta oscillatory power were identified in left temporal and frontal regions during verb generation and corresponded closely to language regions identified during cortical stimulation mapping (see Fig. 1b). Time–frequency analysis of MEG virtual electrodes constructed in left frontal and temporal regions revealed early sustained responses within 200 ms of word onset (see Fig. 1a).

**Fig. 2:**
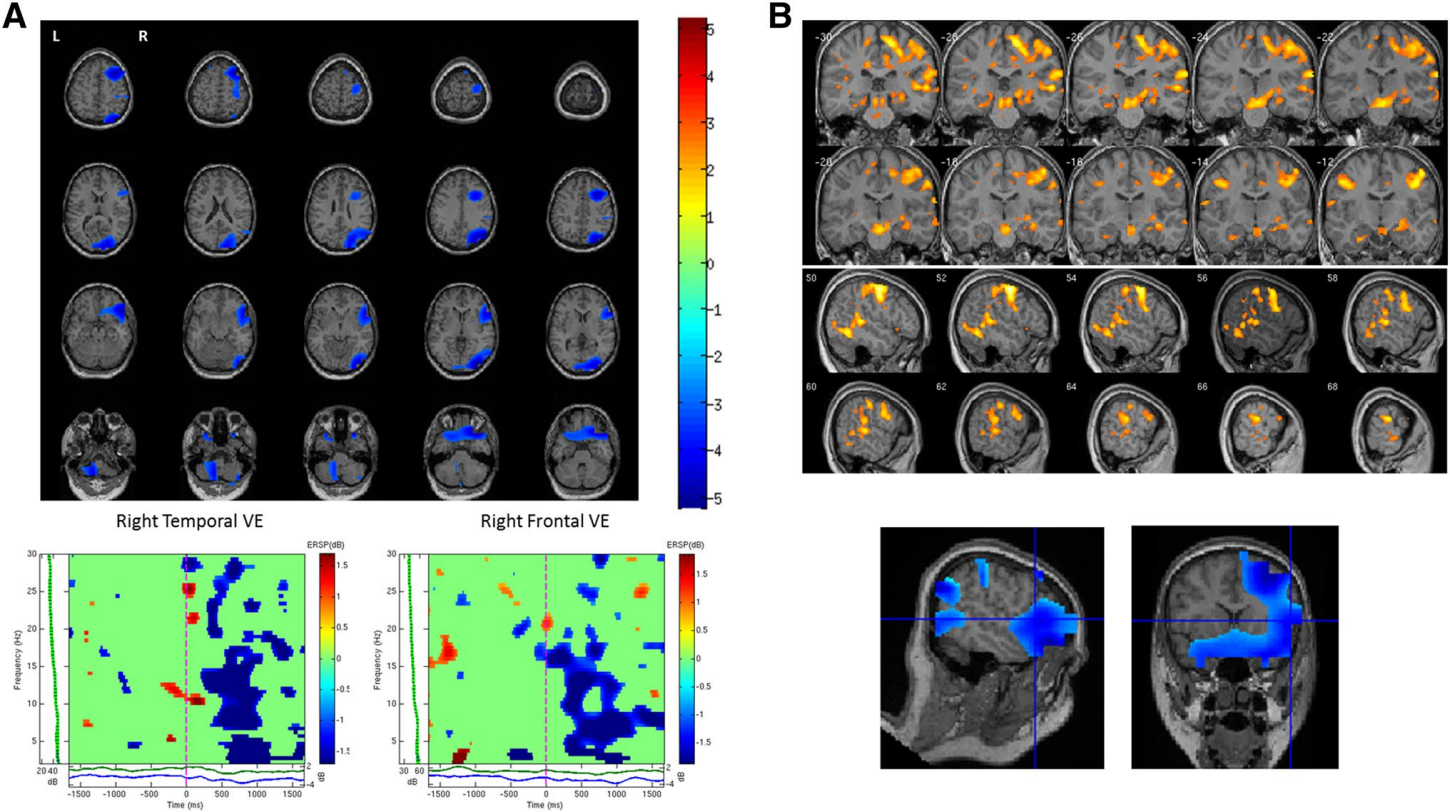
**a** Beamformer source localization results of verb generation task for an individual patient (Pt. 11) are shown overlaid on axial slices of the patient’s T1 MRI. Sources showing significant decreases in beta oscillatory power (13–30 Hz) from 2.5 s pre- to 2.5 s post-stimulus onset were identified in right temporal and right frontal regions (LI = − 47.91). Time–frequency analysis of a virtual electrode (VE) constructed in the right temporal lobe shows significant decreases in beta power from approximately 300 ms post-stimulus onset. Similarly, time–frequency analysis of a virtual electrode constructed in the right frontal lobe shows significant decreases in beta power from approximately 300 ms post-stimulus onset. **b** Sources of decreased beta power identified with MEG correspond with fMRI activation during the same covert verb generation task showing right lateralized activation. fMRI activation is shown overlaid on coronal and sagittal slices of the patients MRI. Blue cross-hairs indicate region of peak decrease in MEG beta power in the right inferior frontal gyrus which corresponds to area of increased activation in the fMRI data

In the remaining eighteen cases where cortical stimulation was deemed not clinically appropriate and therefore not performed, MEG results were compared against a combination of neuropsychological, fMRI and post-operative data as clinically available. In this group, MEG data revealed twelve patients with left hemisphere lateralization, four patients with right lateralization, and two patients with bilateral representation. Eleven out of twelve patients showing left hemisphere lateralization with MEG, were confirmed to be left hemisphere dominant based on the available clinical data. Two out of four patients showing right lateralization with MEG also displayed corresponding right hemisphere dominance in the other clinical data. Figure 2 shows MEG and fMRI data for an individual patient (Pt. 11) who displayed right hemisphere lateralization for language (LI = -47.91). MEG sources of decreased beta oscillatory power (13–30 Hz) were identified in right temporal and frontal regions during verb generation and were consistent with sources of increased activation identified with fMRI during the same task (see Fig. 2b). Time–frequency analysis of MEG virtual electrodes constructed in right frontal and temporal regions revealed early responses within 200 ms of word onset (see Fig. 2a). The two patients who were identified as having bilateral language representation with MEG were concordant with the other clinical data.

With respect to those cases in which there was divergence between the MEG and other available clinical data, one patient (Pt. 14), who was found to have right lateralized language representation with MEG, was described to have left lateralized language based on pre-operative neuropsychological assessment (see Table 1). This patient had a left anterior temporal lobectomy with hippocampectomy and did not display any post-operative language deficits at one-year follow-up relative to their pre-surgical competence. Another case (Pt. 17) was found to be right lateralized with MEG but was described as left lateralized based on neuropsychological assessment. This patient had a right frontal lesionectomy and did not display any post-operative language deficits following surgery. In contrast, patient 25 was found to have left lateralized language activity with MEG but fMRI revealed bilateral language activation (see Table 1). Since this patient did not proceed to surgery due to subsequent improvement in seizure frequency, we were unable to further validate the MEG findings.

Overall concordance between MEG lateralization and other clinical data was found in 25/28 patients. This represents 89% concordance between MEG language lateralization based on beta oscillatory power and a combination of clinical data, including cortical stimulation mapping, fMRI, neuropsychological and post-operative data.

## Discussion

The aim of the present study was to examine feasibility and efficacy of MEG to identify hemispheric dominance for language in a group of pediatric patients in the context of pre-surgical evaluation for drug-resistant focal epilepsy. Our findings have shown that hemispheric dominance for language can be reliably determined with MEG in our sample of children using a verb generation task and beamformer-based spatial filtering. We found significant decreases in beta (13–30 Hz) oscillatory power primarily in the dominant hemisphere that peaked approximately 500 ms after stimulus onset. Spectral analysis within pre-defined regions of interest in bilateral inferior frontal and superior temporal regions were used to further elucidate the temporal progression and oscillatory dynamics within the language network during covert verb generation. This provided the opportunity to assess the level of bi-hemispheric oscillatory dynamics within specified regions of the language network during verb generation.

We found an initial decrease in beta power bilaterally in superior temporal regions, followed by significantly greater sustained power decreases in the dominant hemisphere in fronto-temporal regions. In many cases the non-dominant hemisphere showed increases in beta power after around 500 ms, which is consistent with previous findings in adults (Fisher et al. 2008). Previous language studies in adult epilepsy patients (Findlay et al. 2012; Hirata et al. 2010, 2004; Kim and Chung 2008), healthy adult participants (Passaro et al. 2011) and adolescent participants (Pang et al. 2011) using similar beamformer analysis methods have shown robust decreases in beta oscillatory power in fronto-temporal regions in the dominant hemisphere. Notably some of these studies did not focus on the emerging dynamics of hemispheric dominance during language processing as they examined a single time window only or did not explore the virtual electrode outputs (Hirata et al. 2010, 2004; Pang et al. 2011). Potentially failing to detect the early bilateral activation seen in this study and also reported previously in a verb generation task (Findlay et al. 2012) and during a semantic decision-task (McDonald et al. 2010).

Our motivation to focus on induced oscillatory activity rather than concentrating on evoked activity like many previous clinical MEG studies was guided by increasing evidence on the significant role of neural oscillations in binding and information transfer between brain regions involved in cortical processing (Buzsaki and Draguhn 2004; Engel et al. 2001; Roopun et al. 2008). Beta oscillatory activity has been shown to be a clinically relevant measure of hemispheric dominance in a large cohort of adult epilepsy patients (Hirata et al. 2010). In terms of the functional relevance of neural oscillations during language tasks, beta frequency oscillations have been linked to various aspects of language processing including top-down mechanisms (Weiss and Mueller 2012). In this context, we postulate that decreased beta power in the dominant hemisphere may reflect activation and greater involvement of fronto-temporal regions during language processing, whereas the often observed increases in power on the contralateral hemisphere may represent a measure of the interplay between homologous language-relevant regions (Fisher et al. 2008).

This study was designed as a pragmatic retrospective validation study. We assessed the reliability of our MEG language localization method against other available clinical information. In adult studies the IAP has regularly been used as the gold-standard for language lateralization to validate MEG findings (Findlay et al. 2012; Hirata et al. 2010; Papanicolaou et al. 2004). The relationship with pre-surgical electro-cortical stimulation in the context of chronic invasive recordings performed to confirm pre-operative lateralization hypothesis has been reported only sporadically (Simos et al. 1999b). In these patients, good concordance between MEG and invasive language mapping procedures (75–95%) was seen. However, performing such invasive procedures in pediatric age is not always possible, increasing the difficulty in systematic validation of clinical MEG protocols in this age group. We therefore validated our findings by using a combination of clinical measures that were available, including cortical stimulation mapping, fMRI, neuropsychological and post-operative data where possible. Overall, we found a high level of concordance of 89% between our MEG language and those established using other clinical measures.

Functional MRI, which is a more mature clinical tool, has recently been used as a non-invasive measure to validate MEG mapped language (for review see Balter et al. 2016). Good levels of concordance between the two modalities have been reported when using similar language paradigms (Pang et al. 2011; Wang et al. 2012). There is a growing body of evidence showing that at the neural level, increased BOLD signal is associated with decreased cortical oscillatory power in alpha and beta frequency ranges in MEG (Hall et al. 2014; Zumer et al. 2010). The fact that the brain electro-magnetic signal is a direct measure of neural function makes MEG and EEG ideal non-invasive complementary tools to fMRI particularly when concerns exist of a potential effect of proximity of the anatomical lesion to the activated volume of interest. This issue has been documented in a study on patients with fMRI-critical lesions who showed lower lateralization indices and higher prevalence of discordant language lateralization with IAP test than patients without such lesions (Wellmer et al. 2009). While our study was not designed to address this issue, there are physical grounds to postulate that this should be less of an issue with MEG.

Since in our study invasive data or IAP was only requested based on clinical grounds, we cannot provide systematic validation of our findings that allows comparison with data from other studies in which IAP was performed. However, in light of the evidence supporting the predictive value of presurgical neuropsychological and fMRI data on post-operative language outcome in adults (Binder et al. 2008), we have used them as a useful measure to aid in the validation of MEG language lateralization. In real-life situations, a multimodal approach is still widely used when determining suitability for surgery and our data show that MEG language mapping can provide additional reliable non-invasive information during presurgical evaluation in pediatric epilepsy patients, particularly in younger patients and those with lesions. This is also in line with recent recommendations from the American Clinical MEG Society (Bagic et al. 2017).

We found two cases where our MEG lateralization results did not match the pre-operative neuropsychological assessment data. One patient (Pt. 14) was found to have right lateralized language representation with MEG, but was described to have left lateralized language based on neuropsychological assessment (see Table 1). This patient had a left temporal focal cortical dysplasia and was left handed, factors consistent with having atypical language representation (Berl et al. 2014). Furthermore, this patient proceeded to have a left anterior temporal lobectomy with hippocampectomy and did not display any post-operative language deficits at one year follow-up. Taken together, these factors raise the possibility that this patient had atypical/right language lateralization, consistent with our MEG findings. In cases in which discordant data occur, future studies should include additional sources of information regarding language dominance to better understand the usefulness of MEG in a clinical context.

In another patient (Pt. 17), MEG data showed right hemisphere lateralization but neuropsychological assessment suggested left hemisphere lateralization for language. This patient had tuberous sclerosis complex and an early age of epilepsy onset (age 3). These factors can affect development of the language network and may lead to atypical representation in some cases (Berl et al. 2014). This patient had a frontal lesionectomy to remove a lesion in the right middle frontal gyrus and did not display any post-operative language deficits relative to their pre-surgical status. MEG data showed decreases in beta activity in right inferior frontal and superior temporal lobes, regions outside of the resected area. In the absence of cortical stimulation or fMRI data for this patient it is difficult to draw further conclusions about lateralization in this complex case. While presurgical language and memory assessments in children are useful, they are not highly predictive of postoperative outcome (Lah 2004). Hence the need for a multimodal approach in order to establish language lateralization in children with epilepsy particularly in more complex cases. Notably in our study we found that approximately 21% of patients demonstrated atypical language lateralization based on our MEG analysis, where 4/28 (14%) were right lateralized and 2/28 (7%) showed bilateral language representation. This is consistent with previous work showing that atypical language lateralization occurs in approximately 20–30% of epilepsy patients (Berl et al. 2014; Springer et al. 1999). We also had a relatively high number of left-handed patients in our cohort 6/28 (21%) which may have influenced the results given that left handedness and atypical language representation tend to co-occur in higher frequency than for right-handed individuals. In addition, the age range of patients should also be considered when assessing language lateralization as it has been shown that development is characterized by a shift from bilateral to lateralized activation (Kadis et al. 2011; Ressel et al. 2008). By systematically integrating patient-specific clinical and demographic characteristics with non-invasive neuroimaging data as outlined in the current study, patient-specific profiles can be produced that provide valuable information in the presurgical evaluation process (Balter et al. 2016).

Overall our results revealed broad areas of activation predominantly in frontal (BA 6/44/45/47) and temporal regions (BA 21/22/37) during an expressive verb generation task. Pre-defined regions of interest within frontal (BA 6/44/45) and temporal (BA 22) areas were used to provide an estimate of hemispheric dominance. These specific regions were chosen as they represent areas that are commonly explored with direct cortical stimulation and they are in line with volumes of interest used in previous clinical MEG studies of language in adults (Findlay et al. 2012; Hirata et al. 2004). However, in many surgical cases more precise localization of language areas is required. While this can be inherently difficult due to the complex nature of language processing, it has been shown that the sensitivity and specificity of MEG localization can be increased by using a combination of expressive and receptive language tasks (Findlay et al. 2012). Importantly region of interest analysis is a useful tool in this context, particularly the virtual electrode method described here, as it allows more detailed assessment of the dynamic profiles of language processing within specific regions of the language network. Future MEG studies with larger cohorts of paediatric patients using a combination of receptive and expressive language tasks may provide a means of exploring more precise localizations within the language network.

This study has inherent limitations. Due to its pragmatic nature, a comparison of the tolerability of MEG versus fMRI in this cohort of patients was not possible; future studies to benchmark the two techniques against specific outcome measures might offer some insight in establishing clinical indications for each of the modalities. Furthermore, this study focused on the analysis of changes in MEG spectral power, which are used to detect “local” changes in synchronous activity and provide a good measure of hemispheric dominance. This was driven by the consolidated knowledge of the role of beta oscillations in processing semantic and syntactic aspects of language (Weiss and Mueller 2012) but fails to capture the complexity of network interactions within the language network. In conclusion, our findings suggest that MEG–based measures of language lateralization can provide clinically relevant information in assessing patients from early childhood. Our data suggest that qualitative hemispheric differences in task-related changes of spectral power could also offer a promising insight into the contribution of dominant and non-dominant hemispheres in language processing and may help to characterize the specialization and lateralization of language processes in pediatric age.
